# Complete Genome Sequence of the Nitrogen-Fixing and Solvent-Producing Clostridium pasteurianum DSM 525

**DOI:** 10.1128/genomeA.01591-14

**Published:** 2015-02-19

**Authors:** Anja Poehlein, Alexander Grosse-Honebrink, Ying Zhang, Nigel P. Minton, Rolf Daniel

**Affiliations:** a Genomic and Applied Microbiology and Göttingen Genomics Laboratory, Georg-August University, Göttingen, Germany; b The Clostridia Research Group, BBSRC/EPSRC Synthetic Biology Research Centre, School of Life Sciences, Centre for Biomolecular Sciences, University of Nottingham, Nottingham, United Kingdom

## Abstract

Here, we report on the closed genome sequence of Clostridium pasteurianum DSM 525, which is an anaerobic, Gram-positive and endospore-forming organism. C. pasteurianum can fix N_2_ and produce solvents such as butanol and 1,3-propanediol from carbohydrates. The genome consists of a single 4,350,673-bp replicon.

## GENOME ANNOUNCEMENT

The Gram-positive anaerobic spore-forming bacterium Clostridium pasteurianum DSM 525, is able to produce butanol from carbohydrates (1). In contrast to most other solventogenic clostridia, C. pasteurianum is able to grow with glycerol as sole carbon and energy source (1, 2) and couple glycerol breakdown with a highly active butanol-producing pathway The major products during glycerol degradation are 1,3-propanediol, ethanol, and butanol (1, 3).

Strain DSM 525 was derived from the DSMZ (Braunschweig, Germany). Chromosomal DNA of C. pasteurianum DSM 525 was isolated using the MasterPure complete DNA purification kit (Epicentre, Madison, WI, USA). Subsequently, 454-shotgun and Illumina paired-end libraries were generated from the isolated DNA as described by the manufacturers. The libraries were sequenced using a 454 GS-FLX system (Titanium GS70 Chemistry, Roche Life Science, Mannheim, Germany) and MiSeq Illumina system (Illumina, San Diego, CA, USA), respectively. Sequencing yielded 201,156 454-shotgun and 1,215,244 paired-end Illumina reads. Assembly of the reads using the Roche Newbler assembly software 2.9 and the MIRA software (4) resulted in 139 contigs. For scaffolding and contig ordering, the move contigs tool of the Mauve genome alignment software (5) was used. The closed genome of C. pasteurianum ATCC 6013 (CP009267) served as the reference. Remaining gaps were closed by PCR-based techniques and Sanger sequencing of the products using BigDye 3.0 chemistry and an ABI3730XL capillary sequencer (Applied Biosystems, Life Technologies GmbH, Darmstadt, Germany). For this purpose, the Gap4 (v4.11) software of the Staden package (6) was employed. The complete genome of C. pasteurianum DSM 525 consists of a single chromosome of 4,350,673 bp with an overall G+C content of 30%. Automatic gene prediction was performed with the software tool prodigal (Prokaryotic Dynamic Programming Genefinding Algorithm) (7). Identification of rRNA and tRNA genes was done with RNAmmer (8) and tRNAscan (9), respectively. An integrated microbial genomes/expert review (IMG/ER) system (10, 11) was used for automatic annotation, which was subsequently manually curated by using the Swiss-Prot, TREMBL, and InterPro databases (12). We identified 10 rRNA operons, 81 tRNA genes, 3,220 protein-encoding genes with function prediction, and 768 genes coding for hypothetical proteins. Genes coding for key enzymes of butanol fermentation such as butyryl-CoA dehydrogenase (*bcd*), electron transfer flavoprotein (*eftAB*), 3-hydroxybutyryl-CoA dehydrogenase (*hbd*), and 3-hydroxybutyryl-CoA dehydratase (*crt*) form a cluster that is identical to those identified in other solventogenic clostridia, such as C. acetobutylicum, C. saccharoperbutylacetonicum, or C. saccharobutylicum (13–15). In addition, the genome of C. pasteurianum DSM 25 harbors a cluster coding for CoA transferase (*ctfAB*), acetoacetate decarboxylase (*adc*), and alcohol/aldehyde dehydrogenase (*adhE*), which showed the identical arrangement as the *sol* operon of C. acetobutylicum (16). We also encountered genes encoding acetate kinase (*ackA*), phosphate acetyltransferase (*pta*), butyrate kinase (*buk*), and phosphate butyryltransferase (*ptb*). In addition, the presence of the previously described genes encoding key enzymes for 1,3-propanediol production such as B_12_-dependent glycerol dehydratase (17) and 1,3-propanediol dehydrogenase (2) was confirmed.

### Nucleotide sequence accession number.

The complete genome sequence has been deposited in GenBank under the accession no. CP009268.
